# Performance and clinical usefulness of the Optimal-IT^®^ test in the treatment of confirmed malaria cases in rural areas in Côte d’Ivoire

**DOI:** 10.5281/zenodo.10887947

**Published:** 2014-12-04

**Authors:** Yapo T. Aba, Emmanuel Bissagnené, Ouffoué Kra, Serge B. Assi, Raoul Moh, Pulchérie Goly, Nogbou Ello, Alain Kassi, Bessy R. Yao, Franklin Abouo, Eboi Ehui

**Affiliations:** 1 Département Dermatologie-Infectiologie, UFR Sciences médicales, Abidjan, Côte d’Ivoire; 2 Service des Maladies Infectieuses et Tropicales, CHU de Bouaké, Bouaké, Côte d’Ivoire.; 3 Institut Pierre Richet de Bouaké, Bouaké, Côte d’Ivoire; 4 Centre Médicosocial de la SOGB, San Pedro, Côte d’Ivoire

## Abstract

**Background:**

In Africa, malaria care is mostly based on clinical presumption and the general application of antimalarial treatment to all febrile episodes over several years. Treatment limited to confirmed cases might curb the practice of equating fever with malaria, antimalarial drug abuse and the extension of *Plasmodium* resistance, provided that powerful and reliable rapid diagnostic tests are used. This study aimed at determining the performances of the Optimal-IT^®^ test in the strategy for the exclusive treatment of uncomplicated malaria in rural areas.

**Materials and Methods:**

A prospective study conducted in the forest region of San Pedro, Côte d’Ivoire, included patients exhibiting clinical signs of uncomplicated malaria who gave their consent and benefited from thick blood film (TBF), blood smear (BS) and Optimal-IT^®^ (*pLDH*-based) test. Rapid diagnostic test (RDT) results were taken into consideration to decide on malaria treatment and then compared with TBF/BS results (reference) to assess the performances and clinical usefulness of the RDT.

**Results:**

The mean age of the 384 patients included (209 men, 175 women) was 28 years and the mean temperature was 38.1°C. TBF/BS and Optimal-IT^®^ were concordant in 92% of patients but discordant in 10 false negative (3%) and 19 false-positive patients (5%). The average parasite density of *P. falciparum* was 25,600 trophozoites/μl. The performances calculated were: sensitivity=95%, specificity=91%, positive predictive value=90%, negative predictive value=95%, positive likelihood ratio=10, negative likelihood ratio=0.06 and diagnostic odds ratio=166, indicating that Optimal-IT^®^ is a powerful and credible diagnostic tool. The 193 RDT-positive patients treated were healed, despite three recurrence cases at day (D) D_17_, D_25_ and D_27_, respectively. RDT-negative patients received various treatments (antibiotics, paracetamol), but two patients among them presented with a bout of malaria on D_7_. None of the previously untreated patients returned with severe malaria.

**Conclusions:**

The Optimal-IT^®^ test, which is already used in the field, showed good performances to effectively detect patients with and without malaria. It is therefore adapted to the malaria treatment strategy limited to confirmed cases.

## 1 Introduction

There is currently a renewed interest in the fight against malaria with the support of many initiatives, which raises the possibility of its elimination/eradication. Within this framework and in order to enable endemic countries to steadfastly embark on this perspective, the World Health Organization has made some recommendations to give an orientation to national strategies, such as early care for confirmed cases in health centres and at home [1].

Unfortunately, one of the limitations of this new policy might emanate from diagnostic problems, as some healthcare providers and the general population are used to clinical diagnosis and remain less informed or even reticent about rapid diagnostic test (RDT) results [2,3]. In consideration of this and because of the absolute necessity to improve the diagnosis to reduce antimalarial drug (ab) use, treatment cost and the risk of appearance of *Plasmodium* resistance, the WHO recommends the systematic confirmation by RDT or thick blood film/blood smear (TBF/ BS) for any presumed fever case, and malaria treatment only for febrile patients carrying *Plasmodium* [1]. Thus, in this novel strategy, any clinical presumption must be confirmed before initiating malaria treatment. This indicates that, when the RDT is negative, the physician must refrain from prescribing an antimalarial drug. He/she must rather carry on investigating other causes of febrile illness, from which the patient might also die if not treated in time. However, it appears that some clinicians and other prescribers do not change their prescribing practices when using RDT, with the result that many patients are continuously treated with artemisinin-combination therapies (ACT), despite negative results of RDTs [2,3].

These practices are still a source of ACT wastage and selection for mutant drug-resistant parasites that are already emerging in Asia, with the risk of reaching African countries, as did chloroquine resistance [4,5]. In reality, the concern of reluctant healthcare providers is to determine whether the RDT outcome is reliable enough to enable the decision to prescribe ACT or not. Although some RDTs were initially deemed promising and cost-effective these subsequently showed to be of no or limited interest, owing to the high seasonal variability of *Plasmodium* transmission [6]. Their second concern is to know whether the decision not to treat RDT-negative patients might not expose these patients to severe malaria. The present study aimed to evaluate the performance and clinical usefulness of the Optimal-IT^®^ RDT test in the strategy for the exclusive treatment of confirmed mild malaria in forest rural areas.

## 2 Patients and Methods

### 2.1 Study site

The study was implemented in the Medico-Social Centre (MSC) of the oilseed and rubber Company of Grand-Béréby (SOGB), an agro-industrial complex of 85,775 acres of rubber and oil palm plantation located 80 km from San Pedro city, which lies 348 km from Abidjan. The plantation is surrounded by watercourses and wetlands retaining rainwater. It benefits from dense vegetation, similar to an equatorial forest, as well as a tropical climate with two rainy seasons (April-July, October-November), and two dry seasons (September and December-March). The MSC comprises one central unit (CU) managed by a chief doctor, and 18 village health huts (VHH) run by nurses and community workers. In 2009, malaria accounted for 70% of the reasons for consultation. At the VHH level, where the systematic practice of equating fever with malaria is common, there were cases of clinical malaria. At the CU level, the laboratory performed TBF/BS, but the Optimal-IT^®^ test was performed only in the case of negative TBF/BS (annual activity report 2009). Furthermore, it offered the opportunity to perform other laboratory tests to investigate fever, namely haemogram, chest X-ray, HIV serology, sputum examination for AFB, urine test strips and abdominal-pelvic ultrasonography. The population of this complex was 20,000 inhabitants, of whom 94% were workers at the complex. The internal social security covers 80% of the medical expenses of these workers.

### 2.2 Study population

The target population for this study was febrile patients who attended day consultations between February and September 2010. Patients aged ≥18, with clinical signs suggestive of mild malaria, residing in the vicinity of the MSC, who gave their informed consent and benefited from both RDT and TBF/BS, were enrolled. Conversely, cases of severe malaria, digestive intolerance (diarrhoea and vomiting), antimalarial drug intake within 14 days prior to the consultation, refusal to provide a blood sample, refusal to take the prescribed antimalarial medications or study protocol violation were excluded. According to the Schlesselman formula, 30% is the proportion of patients actually suffering from malaria who may be treated with an appropriate antimalarial treatment. This is based on the results of a previous study of Menan *et al.* [7]; and with a 95% confidence interval (CI), an a-value of 5%, a b-value of 20%, a power (1–b) of 80% and a precision (i) of 5%, the number of patients required (*N*) was estimated at 384, while using a rate of 10% lost to follow-up and study withdrawal.

### 2.3 Methodology

The study evaluated an RDT already in use in the field because of its ability to discriminate between patients with and without malaria. It was prospective, monocentric and was conducted as follows: at the patient selection post (step 1), two nurses arranged the anonymous patient IDs in order of arrival, completed the forms regarding the patients’ age, gender, weight, temperature, height, blood pressure and respiratory rate. At the clinical post (step 2), the study investigator, a PhD student, collected the anamnesis data, medical history, data on patients’ physical examination, performed the Optimal-IT^®^ test according to the manufacturer’s instructions (DIAMED, Cressier, Switzerland), obtained patients’ informed consent and conducted their follow-up. At the sampling post (step 3), blood was aseptically collected from the fourth fingertip of the left hand by a biotechnician to perform TBF/BS. At the phase of therapeutic decision-making supervised by the chief doctor (step 4), RDT-positive patients immediately started antimalarial treatment according to national guidelines. RDT-negative patients, on the other hand, did not receive antimalarial treatment: instead they were re-examined and investigated with other laboratory tests, including TBF/BS, before being treated either with an antimalarial drug if TBF positive, or with another treatment based on symptoms and laboratory test results (antibiotic, antipyretic, vitamins). Thus, TBF and BS results were read *a posteriori* but on the same day as the Optimal-IT^®^ test. At the follow-up phase (step 5), a monitoring was established at the patient recruitment site (CU), village infirmaries (VHH) and at each patient’s home with the assistance of community workers residing in the various farming villages. This always encouraged patients to comply with their follow-up schedule. This rigorous follow-up helped to evaluate treatment efficacy, tolerance, patient compliance and the outcome of all patients included in the study at day (D) D_0-7_, D_14_, D_21_ and D_28_, with TBF/BS retesting in case of fever but without PCR correction for technical reasons.

### 2.4 Regulatory aspects

During their selection, patients were identified by anonymous codes assigned in order of arrival at the MSC at D_0_. They gave their informed consent before being included. They were informed of the objectives and purpose of the study, which aimed to improve their medical care in terms of public health. They benefited from free medical consultations, parasitological research and medicines as part of their social security cover at SOGB. All patients preselected but not included, or those who withdrew from the study, also benefited from adequate care and follow-up in accordance with the study protocol.

### 2.5 Evaluation of Optimal-IT^®^ test

The parasitological results of TBF and RDT were compared in a contingency table by considering TBF as the gold standard (reference). The validity of Optimal-IT^®^ test was determined by the sensitivity (Se), specificity (Sp), positive predictive value (PPV) and negative predictive value (NPV). The clinical usefulness criteria were defined by the positive likelihood ratio (PLR), negative likelihood ratio (NLR) and diagnostic odds ratio (DOR). Consistency between the results of RDT and those of the TBF/BS was assessed by the kappa coefficient ‘κ’. The concordance between the results of the TBF/BS and RDT was considered excellent if ‘κ’≥0.81, good if ‘κ’=0.80-0.61, moderate if ‘κ’=0.60-0.41, mediocre if ‘κ’=0.40-0.21 and poor if ‘κ’<0.20. According to these parameters, a RDT was deemed interesting (powerful and discriminant) and therefore useful in routine practice if the following requirements were met: PLR≥10 or NLR<0.1 and DOR>1 [8,9].

### 2.6 Evaluation of malaria treatment

After inclusion, RDT-positive patients immediately received either artesunate+amodiaquine (ASAQ) or artemether+lumefantrine (AL) for 3 days, oral quinine for 5 days or IM artemether for 7 days. The efficacy of these treatments was assessed from the thermal (T<37.5°C) and parasite (negative TBF) clearance. Thus, the therapeutic response or adequate clinical and parasitological response (ACPR) was defined by the absence of parasitaemia on D_28_, irrespective of axillary temperature, without previously meeting any of the criteria of early treatment failure or late clinical or parasitological failure [10]. Recurrence was referred to as the reappearance of fever and TBF positivity subsequent to an effective malaria treatment. Due to a lack of suitable equipment, no PCR correction was performed to distinguish recrudescence (true parasitological failure) from re-infestation. Compliance was assessed in patients who received at least one dose of treatment (compliance=number of tablets taken/theoretical number of tablets to be taken), which enabled to distinguish compliant patients (compliance=100%) from non-compliant patients (compliance<100%). The intensity of side effects against treatments was classified as grade 0 (absent), grade 1 (mild), grade 2 (moderate), grade 3 (severe) and grade 4 (very severe), according to the WHO classification system [11].

The future conditions of RDT-negative patients, who therefore did not start antimalarial treatment immediately, was determined at D_1-7_, D_14_, D_21_ and D_28_ from clinical data, TBF/BS retesting, other investigation results and treatment prescriptions depending on laboratory tests results.

### 2.7 Statistical analysis

All patients’ data were collected using a standard patient form, then recorded and analysed using the Epi info 6.0 software (CDC/OMS). The distribution of quantitative variables was described by the mean±standard deviation (s.d.). The qualitative variables were described as total number and percentage. The chi-square test and Fisher’s exact test were used to compare the proportions with a 5% significance threshold (*P*<0.05). To assess the accuracy of estimates, we used a confidence interval of 95% (IC_95%_).

The primary endpoint of the study was represented by the likelihood ratios (PLR, NLR and DOR), indicating both the performance and clinical usefulness of the Optimal-IT_®_ test. The secondary endpoints were represented by the proportion of patients who presented with an ACPR at D_28_, the performance criteria (Se, Sp, PPV, and NPV), the thermal and parasite clearance, the recurrence rate at D_3_ and D_28_, the side effect rate and patient adherence to antimalarial medications. The therapeutic response was classified as adequate clinical and parasitological response (ACPR), early therapeutic failure (ETF), late clinical failure (LCF) and late parasitological failure (LPF) in accordance with WHO definitions [10].

## 3 Results

**Figure 1.**
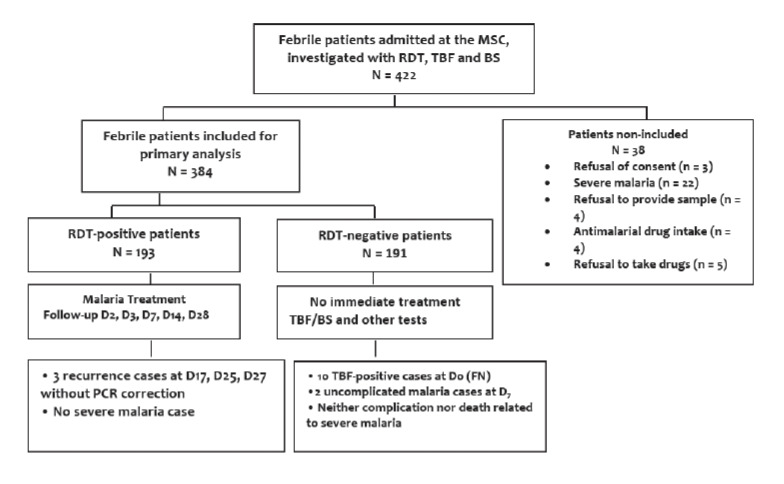
Flow chart of study subjects

In eight months, 422 patients were consulted for febrile episodes suggestive of malaria. Of these, 38 patients were excluded (Fig. 1). Thus, 384 patients were included in the study for primary analysis, of which 347 patients were recruited during the rainy seasons and 37 during the dry seasons of the year. The highest number of patients was recruited in June (54%).

Following the result of the Optimal-IT^®^ RDT, 193 patients immediately initiated malaria treatment. Their follow-up until D_28_ was marked by three recurrence cases at D_17_, D_25_ and D_27_ but without PCR correction. Conversely, the 191 RDT-negative patients did not receive malaria treatment immediately: they were re-examined, investigated and followed up until D_28_. Their follow-up was marked by malaria identification and treatment in 10 TBF-positive patients after confrontation of the two examination results, and two other patients presenting with uncomplicated malaria and positive TBF at D7 of follow-up.

The study sample consisted of 209 men (54.5%) and 175 women (45.5%; Table 1). The age range was between 18 and 67 years with a mean of 38±3.4 years. The majority of patients (73%) were scarcely using efficient prevention methods, such as long-lasting insecticide-treated bednets (LLIN) (30.1%). None of them was splenectomised or transfused. About 6% reported an allergy to 4-aminoquinoline-induced pruritus, and 4.2% were known to be HIV-1-positive without receiving antiretroviral therapy. No significant relationship was observed between the presence of malaria and the demographic and seasonal parameters when the double-positive cases (TV=174) and double-negative cases of the tests (TN=181) were compared.

**Table 1.** Relationship between socio-demographic characteristics and malaria

Parameter	Malaria present *N* = 174	Malaria absent *N* = 181	OR	CI	P
*Season*			1.02	0.51-2.08	0.96
Rainy season	156	162			
Dry season	18	19			
*Age (years)*			NA	NA	NA
≤ 50	170	181			
> 50	4	0			
*Gender*			0.99	0.66 -1.51	0.98
Male	95	99			
Female	79	82			
Prevention method*			1.02	0.65 -1.61	0.91
Used	122	126			
Not used	52	55			

NA: not applicable

* long-lasting insecticide-treated bed nets (LLIN), serpentin, insecticide spray, repellent cream/lotion, intermittent preventive treatment with sulfadoxine-pyrimethamine (IPT-SP)

### 3.1 Clinical and biological features of malaria

The clinical manifestations variously associated fever (100%), dark urines (86%), headaches and arthralgias (70%), body pains (62%) and splenomegaly (8.3%). The mean temperature was 38.1±0.8°C (range: 37.8-40.1°C) and the mean weight was 62.3±12.8 kg (range: 47-82 kg). *P. falciparum* was identified at D_0_ with a mean parasite density of 25,600 trophozoites/μl (range: 50,000-106,000 tr/μl). Nevertheless, the positivity rates of the two tests (TBF, RDT) gradually increased in proportion to the rise in parasite density (Table 2). No gametocytes were detected.

**Table 2.** Parasite density of *Plasmodium falciparum* based on diagnostic method

Parasite density (trophozoites/μl)	TBF positive *N* = 184	RDT positive *N* = 174
<100	7 (3.8)	7 (4.0)
100-500	15 (8.2)	9 (5.2)
500-1000	18 (9.8)	12 (6.9)
>1000	144 (78.3)	146 (83.9)

### 3.2 Credibility and clinical usefulness of Optimal-IT^®^ test

The proportion of malaria-positive patients was 48% (CI: 43-52%) with TBF (*a posteriori* probability to detect malaria), 50% (CI: 45-55%) with Optimal-IT^®^ (*a priori* probability to detect malaria) and 45% (CI: 40-50%) with their double positivity (Table 3). Thus, Optimal-IT^®^ positivity increased the probability to detect malaria by 2%. The two tests were concordant in 92% of patients with κ=0.64.

**Table 3.** Optimal-IT performances for malaria diagnosis compared to thick blood film (TBF)

Index test	Clinical condition and TBF positive (malaria present)	Clinical condition and TBF negative (malaria absent)	Total
RDT positive	174 (True Positive)	19 (False Positive)	193
RDT negative	10 (False Negative)	181 (True Negative)	191
Total	184	200	384

Considering the performance criteria (Se=95%, Sp=91%, PPV=90%, NPV=95%) and clinical usefulness (PLV=9.96, NLR=0.06, DOR=166), Optimal-IT^®^ test was more often positive in patients suffering from malaria than in patients without malaria. Thus, in the present analysis, 166 RDT-positive patients, effectively having malaria, were detected against one RDT-positive patient not having malaria.

The Optimal-IT^®^ test results were available within 15-20 min (mean 17±2.0 min), whereas the result turnaround time for microscopy ranged between 45 and 300 min with a mean of 100±20 min. Thus the diagnosis time limit for RDT was substantially shorter than that of TBF.

Resorting to Optimal-IT^®^ test helped avoiding the systematic prescription of antimalarial drugs to 191 RDT-negative patients, which corresponds altogether to a saving of antimalarial drug use of about 50% out of all included patients. However, compared with TBF, RDT over-detected 19 patients (false positives), namely a 9.8% calculated risk of additional antimalarial drug use. On the other hand, it did not detect 10 TBF-positive patients (false negatives), representing a 5.2% calculated risk of absence of antimalarial treatment (Table 3).

During follow-up of RDT-positive patients undergoing malaria treatment, the number of medical consultations was two-fold reduced compared with the group of RDT-negative patients. However, when the RDT was negative, laboratory tests, X-rays and antibiotics were prescribed significantly more often than in RDT-positive patients. The duration of patients work stoppage, which is known and assessable in 250 workers (70 RDT-positive patients versus 180 RDT-negative patients), varied significantly between the two groups of patients analysed. Finally, neither complication nor death in relation to severe malaria occurrence was observed in the two groups of patients analysed.

In total, 205 patients (193 RDT-positive patients, 10 TBF-positive patients at D_0_ and 2 TBF-positive patients at D_7_) were treated with ASAQ (154 cases), AL (39 cases), oral quinine (6 cases) and IM artemether (6 cases). Clinical and parasitological healing was observed in all cases, with apyrexia and parasite clearance at D_2_-D_3_ in 194 patients (94.6%) and at D_4_-D_5_ in 11 patients (5.4%). However, three recurrence cases were observed at D_17_, D_25_ and D_27_ among the 154 patients treated with ASAQ (2%). These three patients were successfully re-treated, the first with AL and the two others with ASAQ.

During follow-up, no patient of this group developed severe malaria but 45 patients reported adverse reactions against antimalarial drugs (22%): 35 patients on ASAQ (23%), 6 on AL (15%), 2 on oral quinine (33%) and 2 on IM artemether (33%). Abdominal pains (17 cases), asthenia (12 cases), general discomfort (7 cases), nauseas (8 cases), pruritus (3 cases), dizziness (2 cases) and pain at the IM injection site (1 case) were reported and deemed mild or moderate in terms of severity. A complementary analysis carried out according to the time limit for starting treatment, the type of treatment and the true positive and false positive profiles of patients did not show a significant difference for the treatment initiation time limit or type of treatment. Conversely, the proportion of patients with adverse reactions was significantly higher among false-positive patients (*P*=0.001).

With regard to compliance, 4 patients (2%) had compliance problems at D_2_ (one quinine and AL intake missed) and 6 patients were reported lost to follow-up at D_28_ (3.1%). Finally, among the 181 RDT-negative patients, 179 febrile, non-parasitaemic patients were variously treated in a probabilistic manner, either with antibiotic therapy for presumed bacterial infections (99 cases), or with paracetamol+vitamin C and other drugs for flu-like syndrome (80 cases), as shown in Table 4. No patient returned on D_28_ with severe malaria or a complication related to the disease treated.

**Table 4.** Conditions recorded and treated for RDT-negative patients

Disease	Treatments	Patients treated *N*= 179	%
Bronchopneumonopathy	Amoxicillin	40	22.3
Pharyngitis	Amoxicillin	10	5.6
Urogenital infection	Ciprofloxacin	9	5.0
	Amox+Clavulanic acid	20	11.2
Digestive infection	Pefloxacin	16	8.9
Cutaneous infection	Spiramicin	4	2.2
Flu-like syndrome	Paracetamol	80	44.7
	Expectorant	64	35.7
	Nasal decongestant	66	36.9
	Vitamin C	33	18.4

## 4 Discussion

Constraints with microscopic diagnosis and the risk of therapeutic delay show that resorting to more rapid tools usable within the framework of the novel treatment strategy limited to confirmed malaria cases is necessary and urgent in sub-Saharan Africa. Here, we implemented a study to assess the ability of the Optimal-IT^®^ test to support this strategy also as a means to reduce antimalarial drug use in a rural medical centre where, until 2010, this test was performed only in patients with negative microscopy results.

Although children remain the main victims of malaria in Côte d’Ivoire, the study was intentionally limited to adults (due to constraints of side-effect relationships). Thus, the typical patient of our study was a 38-year-old man working at SOGB, thus residing in a forested and rural area where he scarcely protects himself with a LLIN in spite of proven efficacy [3]. Nevertheless, in the case of mild malaria, he benefits on the spot, either from ACT or oral quinine/IM artemether mono-therapy, according to national guidelines. This treatment is generally well tolerated and effective, with a low rate of recurrence or even recrudescence (estimated to be <5%), thus corroborating the results corrected by PCR [12,13]. Conversely, in the event of severe malaria, the patient is immediately transferred to the regional hospital of San Pedro city.

We noticed that, among patients presenting with typical clinical features of uncomplicated malaria (fever, dark urine, diffuse aches and digestive disorders), 48% had a positive TBF and 50% a positive RDT. In other words, 50-52% of patients were not *Plasmodium* carriers, but they would have received malaria treatment based on clinical diagnosis. These results corroborate the findings of other studies, particularly those of Menan *et al.* [7], who showed that in Abidjan among 605 patients presenting with fever (84%), asthenia (52%), body pains (38%) and vomiting (36%), 70% did not carry parasites but still received malaria treatment. This was not only useless, but also added risk of having masked the presence of other serious diseases [7,14]. At the parasitological level, although *P. malariae* and *P. ovale* are responsible for 1-8% of infections in this region [15], *P. falciparum* is the only species isolated in the present study, with a mean parasite density comparable to those from other studies conducted among native adults [16]. Conversely, in non-immune subjects (children, expatriates), this density is much higher, with the risk of appearing as a poor prognostic factor [17].

The various figures obtained from the present study indicate that malaria remains holoendemic in the forest region of the country. The prevalence of mild malaria is 45% on average, ranging from 48% with the reference test to 50% with Optimal-IT^®^ test. This average is well below the *Plasmodium* ratio observed in rural, moist savannah regions, namely 68% in Allokokro village [18] and 85% in the forest region of Taï [15], but much higher than the 10% rate observed in Abidjan among patients infected with HIV [16]. All these differences confirm that the transmission risk of *Plasmodium* varies according to the population age, immune status, study sites, climate changes, diagnostic methods and the urbanization level, factors already revealed elsewhere [19-23]; in Brazzaville in particular, where we have noticed transmission to vary strongly from one area to another, ranging from less than one infectious bite per person every two years to more than hundred infectious bites per year [24].

Literature data have clearly demonstrated that the systematic application of parasitological research can considerably reduce the prevalence of presumptive or clinical malaria, which is an important factor for reducing the number of useless treatments and therefore malarial drug abuse and the possible risk of selecting resistant parasites [7,25-28]. The results of this study, as in previous studies mainly conducted in urban hospitals, show the overriding importance of laboratory diagnosis, more particularly rapid diagnostic tests, in rural areas. Indeed, they confirm that diagnostic uncertainty related to clinical presumption brings about additional antimalarial prescription that can account for 30-50% in Côte d’Ivoire [7]. Thus, supposing that TBF remains the reference parasitological test, one has to admit that this decrease in prevalence according to clinical presumption is due to Optimal-IT^®^ test use, which improves TBF positivity with low rates of false negatives and false positives [21,25-28]. Indeed, the large-scale use of RDT might incur considerable costs through the prescription of antimalarials to false positives [29,30]. This seems legitimate, but this drawback must be balanced by the advantages of this diagnostic tool, which leads to early treatment initiation, the reduction of the prescription of paraclinical examinations, antimalarials, antibiotics and other unjustified first-line drugs, and a decrease in the number and duration of work stoppage among workers. This highlights the utmost importance of laboratory diagnosis, as this alone can provide information about the evolution of indicators, in this case, malaria incidence, in order to control this disease better [22,23,28].

The results obtained with TBF and Optimal-IT^®^ are concordant, as there is no significant difference between the proportions of bouts of malaria diagnosed by these two methods (*P*>0.05). Considering TBF as the reference, Optimal-IT^®^ has a great capability to identify patients suffering from malaria (Se=95%, PPV=90%) and to discard patients not suffering from malaria (Sp=91%, NPV=95%) with a low error risk (3-5%) as opposed to TBF. These values are close to those observed in Burkina Faso by Valea *et al.* [31], in DRC Congo by Muhindo *et al.* [32] and in Cameroon by Tahar *et al.* [33]. Conversely, in Uganda, Jelinek *et al.* [34] have observed a Se=59% and a Sp=62%. These differences could be explained by the manufacturing quality and/or plate reading but it should be remembered that all abilities of RDT increase with parasitaemia and malaria prevalence [23,26-28,35]. This confirms the results of the present study, in which Optimal-IT^®^ performances were better when parasitaemia exceeded 500 parasites/μl (Table 3). Mindful of the Se, Sp, PPV and NPV variations according to malaria prevalence, we have further determined other criteria in this study, which offer many advantages irrespective of the prevalence of the disease [13,36]. These are the likelihood ratios and diagnostic odds-ratio, which helped to assess the clinical usefulness of Optimal-IT^®^. The results for these criteria, PLR=10, NLR=0.06 and DOR=166, clearly indicate that Optimal-IT^®^ is endowed with interesting and well-performing capacities in terms of discrimination and clinical benefit. Indeed, in this study, there were 166 RDT-positive patients presenting with malaria versus 1 RDT-positive patient not presenting with malaria. Therefore, these results should reassure physicians who still hesitate to rely on RDT outcome in therapeutic decision-making, while acknowledging that they are easy and fast to perform [3,37]. Thus, the results of this study allow us to state that, when the Optimal-IT^®^ result is positive, the probability that the febrile patient has malaria is high, which authorizes the clinician to initiate treatment quickly. By doing so, he/she indeed takes the risk of needlessly treating false positives, due to several factors, such as very late reading after depositing reagents, the presence of auto-antibodies or rheumatoid factor [25,28-30]; however, this false-positive risk is relatively low (<5%), which constitutes a factor of low antimalarial drug use and treatment cost [38-40]. On the other hand, in view of the evolutive data of RDT-negative patients, Optimal-IT^®^ negativity can lead the physician to refrain from prescribing malaria treatment immediately, especially to native adults, among whom false negative results, in reduced number, appear to be caused by low parasitaemia. Nevertheless, the febrile patient must be re-examined and investigated with other laboratory tests, including TBF and BS, followed up and evaluated necessarily at D_3_, D_7_, D_14_ and D_28_. The non-immediate prescription of malaria treatment to native RDT-negative patients would not necessarily expose these to severe malaria complications and death [28].

## 5 Conclusions

This study shows that *P. falciparum* remains the primary species of malaria in the forested area of the western part of Côte d’Ivoire. The Optimal-IT^®^ is a technically powerful test to effectively discriminate patients with and without malaria. Its first-line use offers numerous advantages, such as a good concordance with the reference test, the reduction of excessive and abusive use of antimalarial drugs and other unjustified medications. Moreover, the non-treatment of native RDT-negative adults does not expose them to the risk of serious malaria complications. Therefore, Optimal-IT® has proven capable and useful for supporting the treatment strategy limited to the confirmed malaria cases in this study area, where the conditions for microscopy-based diagnostics are not always fulfilled.
